# Serious haematological toxicity during and after ipilimumab treatment: a case series

**DOI:** 10.1186/1752-1947-8-240

**Published:** 2014-07-01

**Authors:** Ester Simeone, Antonio Maria Grimaldi, Assunta Esposito, Marcello Curvietto, Marco Palla, Miriam Paone, Nicola Mozzillo, Paolo Antonio Ascierto

**Affiliations:** 1 Unit of Melanoma, Cancer Immunotherapy and Innovative Therapy, Istituto Nazionale Tumori Fondazione “G. Pascale”, Via Mariano Semmola, 80131 Napoli, Italy; 2 Department Melanoma and Soft Tissue Cancer, Istituto Nazionale Tumori Fondazione “G. Pascale”, Napoli, Italy

**Keywords:** Anaemia, CTLA-4 blockade, Immune-related adverse events, Immunotherapy, Ipilimumab, Leukopenia, Toxicity

## Abstract

**Introduction:**

Immunotherapy with the anti-cytotoxic T-lymphocyte antigen-4 monoclonal antibody ipilimumab has been shown to improve overall survival in previously treated and treatment-naïve patients with unresectable stage III or IV melanoma. Consistent with its proposed immunomodulating mechanism of action, the most common toxicities associated with ipilimumab therapy are immune-related in nature and include those related to the skin and gastrointestinal tract, with endocrine and hepatic events also frequent. Other rare adverse events, including haematological aberrations, may also occur and can have serious consequences if unrecognised. Here we describe three patients who developed serious haematological adverse events during or after treatment with ipilimumab.

**Case presentation:**

Three Caucasian patients (two women aged 68 and 49 years and one man aged 70 years) with metastatic melanoma experienced anaemia and/or leukopenia (neutropenia) with toxicity of various grades during or after treatment with ipilimumab, without significant changes to other haematological values. Two of the patients stopped treatment after the third ipilimumab dose, one because of severe anaemia that required blood transfusion and the other due to febrile neutropenia that was treated with antibiotics and granulocyte-macrophage colony-stimulating factor stimulation. The third patient developed anaemia and leukopenia after treatment during the follow-up period. The results of autoimmunity tests performed were positive and corticosteroids were used to treat these events as per side-effects treatment algorithms specifically developed for the management of immune-related adverse events associated with ipilimumab, an approach that was safe and effective.

**Conclusions:**

Haematological toxicity is a rare but potentially serious immune-related side effect of ipilimumab therapy. However, if promptly recognised and treated, haematological toxicity is manageable and can be reversed with standard corticosteroid treatment as recommended for other ipilimumab immune-related side effects.

## Introduction

Immunotherapy with the anti-cytotoxic T-lymphocyte antigen-4 (anti-CTLA-4) monoclonal antibody ipilimumab has been shown to improve overall survival in previously treated and treatment-naïve patients with unresectable stage III or IV melanoma [1,2]. As a result of these findings, ipilimumab has become a new standard of therapy for metastatic melanoma, being approved as first- and second-line treatment in Europe, the USA and elsewhere. However, because CTLA-4 plays a key role in regulating tolerance to self-antigens, ipilimumab can result in autoimmune damage of various organ systems, leading to sometimes life-threatening or fatal immune-related adverse events. Increasing use of ipilimumab in clinical practice has resulted in improved knowledge of its safety profile with the most frequent toxicities now well known, in particular cutaneous, gastrointestinal, endocrine and hepatic side effects [3]. Other less common adverse effects can also occur. For instance, a recent review of ipilimumab-related adverse events in 702 patients included previously unreported toxicities such as drug rash with eosinophilia and systemic symptoms, ischemic gastritis, granulomatous inflammation of the central nervous system, and aseptic meningitis [4]. In addition, although uncommon, haematological toxicities have also been previously documented, with reports in the literature of anaemia and neutropenia associated with ipilimumab therapy [5-7]. If unrecognised and not promptly treated, these side effects can have serious consequences. Here we describe three patients who participated in the Italian Expanded Access Programme (EAP) [8] and who developed serious haematological adverse events with ipilimumab at the recommended dose of 3mg/kg.

## Case presentation

### Case report 1

A 68-year-old Caucasian woman presented with metastatic disease of an unknown primary melanoma, with disease characterised by in-transit lesions that were treated with repeated surgery. Six years later disease progression comprising a bilateral renal mass was diagnosed, at which time she started chemotherapy with temozolomide. After progression 1 month later, she was started on ipilimumab 3mg/kg. There were no significant side effects for the first three ipilimumab doses. However, before the fourth dose, she experienced symptoms of fatigue and mild dyspnoea. A blood test showed an acute decrease in her haemoglobin (Hb) level, with a value of 6.0g/dL. She was discontinued from the EAP and hospitalised. Cross-testing for blood transfusion showed positivity; anaemia of autoimmune origin was suspected. This diagnosis was confirmed through laboratory evaluation involving: haematology tests (haematocrit 16%; white blood cell (WBC), 9200/μL; platelets 235,000/μL) with reticulocyte evaluation (elevated, 0.2% with absolute count 1.9K/μL); assessment of serum iron (normal, 215μg/mL), serum ferritin (high, 900ng/mL), total bilirubin (elevated, 2.5mg/dL, with indirect fraction also high, 1.5mg/dL), and lactate dehydrogenase (elevated, 580U/L); peripheral blood smear (spherocytes and normal WBCs and platelets); and a positive direct Coombs test. A bone marrow biopsy was not considered necessary in this case, given that her WBC and platelet counts were normal and so not suggestive of bone marrow invasion and because other diagnostic tests were sufficient to indicate haemolytic autoimmune anaemia. Due to this diagnosis, she was started on high-dose methylprednisolone (125mg twice a day) before a selective blood transfusion was performed with washed red cells. Her Hb level increased by 2g/dL in the first 24 hours and then progressively over the course of a week. High-dose corticosteroids were continued until her Hb level increased and was maintained at the higher level for 1 week; corticosteroids were then tapered over a 4-week period. She was monitored with a haematology test once a week for the first month. Her Hb returned to normal ranges without showing any other alterations. Tumour assessment by a computed tomography (CT) scan showed stable disease that has been maintained until the present time (December 2013).

### Case report 2

A 49-year-old Caucasian woman presented with a diagnosis of stage IIIC melanoma of the trunk. The first evidence of disease progression was detected 7 months later with metastases to her bone and brain. A bone marrow biopsy showed lymphocyte infiltration without evidence of other malignant cells. Chemotherapy with fotemustine for two cycles was initiated, but she developed progressive metastatic disease to her brain. After a further 2 months, she underwent CyberKnife® radiosurgery, after which she started ipilimumab at 3mg/kg. After the third dose of ipilimumab, she developed severe leukopenia and neutropenia (WBC count 1×10^3^/μL, neutrophils 1%) with fever (39°C). Blood tests showed no infection and other haematological values were normal. She was promptly hospitalised and treated with antibiotics, granulocyte-macrophage colony-stimulating factor (GM-CSF) and high doses of intravenous methylprednisolone (2mg/kg twice a day) until improvement and resolution after 10 days. Her methylprednisolone dose was tapered over 8 weeks. Subsequent brain magnetic resonance imaging and whole-body CT scans showed a partial response in her brain and bone. She maintained this partial response with normal blood tests for 13 months, after which rapid brain disease progression occurred and she died.

### Case report 3

The third patient was a 70-year-old Caucasian man who received a diagnosis of ocular melanoma, with the first recurrence to laterocervical lymph nodes being excised after 2 years. After a further 2-year period, he developed bilateral lung metastases and started chemotherapy with fotemustine for three cycles, with stable disease for 2 years. Progressive disease metastatic to his lungs was then diagnosed and he received ipilimumab 3mg/kg for four cycles, with stable disease after 12 weeks. The only side effect of ipilimumab was mild pruritus that resolved itself after treatment was complete. At week 48 (from initiation of ipilimumab), assessment of his tumour showed a complete response. During follow-up, he developed severe anaemia (Hb 7.0g/dL) and leukopenia (WBC 900/μL, neutrophils 32%) without fever. Tumour restaging still showed a complete response. A bone marrow biopsy was performed and showed lymphocyte infiltration without evidence of other malignancies or melanoma cells while a peripheral blood smear showed an absence of blasts or fibrosis and normal cellularity. His WBCs and red blood cells (RBCs) were normal even though CD4 and CD8 lymphocytes were increased in both bone marrow and peripheral blood. No cytogenic alterations were found and no haemolysis was observed. He was successfully treated with oral corticosteroids (prednisone 1mg/kg/day as per side-effects treatment algorithm) and GM-CSF for 1 week. His haematological test results returned to normal and prednisone was tapered over 4 weeks. To date, he maintains a complete response with no other haematological alterations.

## Discussion

Haematological toxicity may potentially involve all blood cell types due to autoimmunity, but the three patients described here have anaemia and/or leukopenia (neutropenia) with toxicity of various grades without significant changes to other haematological values. Two of the patients stopped treatment after the third ipilimumab dose, one because of severe anaemia that required blood transfusion and the other due to febrile neutropenia that was treated with antibiotics and GM-CSF stimulation. The third patient developed anaemia and leukopenia during the follow-up period.

**Figure 1:**
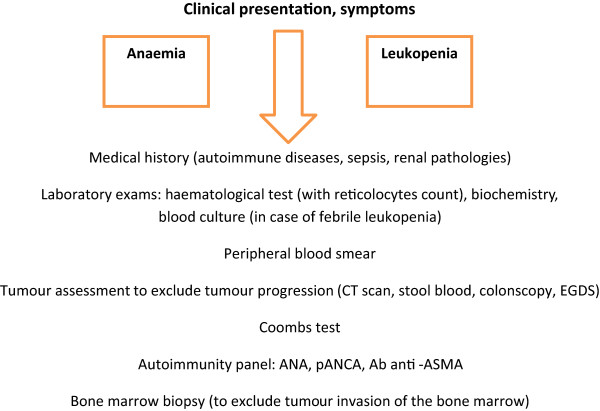
**Work-up for suspected autoimmune anaemia and leukopenia.** ANA, anti-nuclear antibodies; pANCA, anti-neutrophilic cytoplasmic antibodies; Ab anti-ASMA, anti-smooth muscle antibodies; EGDS, esophagogastroduodenoscopy.

In patients who develop haematological toxicity during treatment, the first steps are to exclude other causes through bone marrow biopsy to assess metastatic involvement or other primary malignancies, autoimmunity tests (anti-nuclear, anti-cardiolipin and anti-thyroglobulin antibodies) and haemolysis tests (reticulocytes, Coombs tests). A CT scan and blood stool analysis are also necessary to exclude disease progression. A severe acute reduction in Hb, reduction in haematocrit and RBC levels with an increase in reticulocytes, elevated indirect bilirubin and a positive direct Coombs test are all suggestive of autoimmune anaemia. Our suggested work-up for suspected autoimmune anaemia and leukopenia is shown in Figure 1. All three of these patients were hospitalised and studied to exclude other causes for haematological changes. The results of autoimmunity tests performed were positive and corticosteroids were used to treat these events as per side-effects treatment algorithms specifically developed for the management of immune-related adverse events associated with ipilimumab [9]. High doses of intravenous methylprednisolone at 1 to 2mg/kg twice a day (in case of grade 4 or severe toxicity) or 1mg/kg a day of oral prednisone (in case of grade 3 toxicity) are the recommended corticosteroid choice with the support of antibiotics, GM-CSF and blood transfusion. This approach has been shown to be effective and safe.

A possible explanation for this autoimmune anaemia is that ipilimumab increases the production of auto-antibodies against RBCs, as has been observed in some patients receiving another anti-CTLA-4, tremelimumab [10]. In two previous cases in the literature that describe anaemia [5] or neutropenia [6] associated with ipilimumab treatment, both reported aplasia/hypoplasia of cells in bone marrow biopsy with ipilimumab-related pathogenesis thought to be due to lymphocyte infiltration. Although we observed lymphocyte infiltration in two of our patients (cases 2 and 3), no aplasia/hypoplasia was detected. Moreover, unlike our patients, these previously reported cases were not responsive to corticosteroids. In our third case, the patient had no haemolysis and anaemia was normochromic and normocytic with a negative Coombs test. Thus, we suggest that ipilimumab-related haematological toxicity may be mediated by different autoimmunity mechanisms that result in different toxicities (for example the development of RBC auto-antibodies causing anaemia with a positive Coombs test or ipilimumab-stimulated lymphocyte infiltration of the bone marrow, resulting in a lymphocyte ‘traffic jam’ block with subsequent lymphopenia with or without anaemia).

Immune-related adverse events associated with CTLA-4 blockade typically occur during treatment within 12 weeks of initial dosing [11]. The occurrences of haematological toxicities in two of our patients were consistent with these findings, with onset after the third ipilimumab dose. However, our third case indicates the possibility of a late or delayed toxicity that can occur a considerable time after cessation of treatment. This suggests there is a need to continue complete blood count assessments during long-term follow-up of ipilimumab-treated patients.

Early ipilimumab trials suggested an association between development of immune-related adverse events and clinical response [12]. Although no such correlation was seen in the Italian EAP, with disease control rates of 35.3% in patients with and 33.9% in those without immune-related side effects [13], all three patients with haematological toxicity described here had a response to ipilimumab.

## Conclusions

In conclusion, these reports suggest that, although uncommon, haematological toxicity can occur both during and after treatment with ipilimumab. If promptly recognised and treated, this toxicity is manageable and can be reversed with standard corticosteroid treatment as recommended for other ipilimumab immune-related side effects. As such, clinicians should be aware of the possibility of these less frequent types of immune-related adverse events with ipilimumab.

## Consent

Written informed consent was obtained from the patients (case 1 and 3) and patient’s next-of-kin (case 2) for publication of this case report and any accompanying images. A copy of the written consent is available for review by the Editor-in-Chief of this journal.

## Abbreviations

CT: Computed tomography; CTLA-4: Cytotoxic T-lymphocyte antigen-4; EAP: Expanded Access Programme; GM-CSF: Granulocyte-macrophage colony-stimulating factor; Hb: Haemoglobin; RBC: Red blood cell; WBC: White blood cell.

## Competing interests

ES received honoraria from Bristol Myers-Squibb. PAA received research funding from Bristol Myers-Squibb, Roche-Genetech and Ventana. He also has/had a consultant or advisory role for Bristol Myers-Squibb, Roche-Genentech, Glaxo SmithKline, Ventana and Novartis. He received honoraria from Bristol Myers-Squibb, Roche-Genentech, and GlaxoSmithKline. All the other authors have no competing interest.

## Authors’ contributions

ES, AMG, AE, MC, MaP, MiP and PAA performed all clinical analyses. ES, AMG, and NM helped to draft the manuscript. ES and NM participated in the design of the study. PAA conceived of the study and drafted the manuscript. All authors read and approved the final manuscript.
